# Containment control of multiple unmanned surface vessels with NN control via reconfigurable hierarchical topology

**DOI:** 10.3389/fncom.2023.1284966

**Published:** 2023-10-19

**Authors:** Wei Liu, Fei Teng, Huiyu Xiao, Chen Wang

**Affiliations:** ^1^School of Navigation, Dalian Maritime University, Dalian, China; ^2^College of Marine Electrical Engineering, Dalian Maritime University, Dalian, China

**Keywords:** containment control, neural network control, topology reconfiguration, hierarchical communication topology, LOS guidance

## Abstract

This paper investigates the containment control of multiple unmanned surface vessels with nonlinear dynamics. To solve the leader-follower synchronization problem in a containment control system, a hierarchical control framework with a topology reconfiguration mechanism is proposed, and the process of containment control is converted into the tracking of a reference signal for each vessel on its respective target heading by means of the light-of-sight (LOS) guidance. In a control system, the neural networks (NNs) are adopted to consider the uncertainty. In the follower layer, a connectivity controller with a topology reconfiguration mechanism is embedded, to change the converging positions of followers so as to avoid collision within the system, and to maintain the system connectivity when the communication equality is poor. The effectiveness of the hierarchical control framework proposed in this paper is valid by simulation.

## 1. Introduction

Containment control (Cao and Ren, 2009; Meng et al., 2010) refers that, with the framework of multi-leader-multi-follower system, all the followers are driven by a control protocol so as to converge to the convex hull spanned by all the leaders. A significance of studying the containment control problem is that agents equipped with various advanced sensors can detect a variety of environmental information, including obstacles, etc., during the movement process, and complete the information fusion through distributed algorithms, whereby a dynamic security area is formed. Under the containment control protocol, the followers are able to converge to the safe zone and keep synchronized movement with the leader, achieving cooperation and guaranteeing system safety at the same time. In research aimed at multi-agent systems, containment control has yielded very rich research results (Li et al., 2013; Ma et al., 2015; Wen et al., 2016; Wang et al., 2020).

However, when the application background of the problem changes to marine, many theoretical results in multi-agent systems cannot be well applied. The reasons behind this phenomenon mainly result from the dynamics of the marine crafts, one is that the dynamic is complex, and it is difficult to deal with the strong nonlinearity and strong coupling, and the other is that the dynamic uncertainty is difficult to be known and describe. In motion control problems, there are two commonly used models for the dynamics of vessels: the model derived by Fossen (1994, 2002) and the Nomoto model (Banazadeh and Ghorbani, 2013). The three-degree-of-freedom model derived by Fossen is widely used in various types of problems because it includes kinematics-related state variables. However, the controller form is more complicated and often with higher-order derivative terms. The Nomoto model is usually applied to problems related to heading tracking because it establishes the transfer function relationship between rudder angle and heading. The controller form is simple and has fewer parameters to be determined, but the model accuracy is lower than that of the model derived by Fossen. When used in combination with guidance methods such as line-of-sight(LOS) guidance (Fossen et al., 2015), predictor-based line-of-sight(PLOS) guidance (Liu et al., 2016), adaptive line-of-sight(ALOS) guidance (Gu et al., 2017), etc., the controller form designed based on the Nomoto model is the simplest one. For the uncertainty, the neural networks(NNs) have been applied effectively due to their distinct advantages in recognizing unknown nonlinearity (Li et al., 2020, 2023; Ding and Wang, 2022; Ding et al., 2022; Shao et al., 2022; Teng et al., 2023; Zhou et al., 2023). In (Li et al., 2010), the radial basis function neural network (RBFNN) is used to consider the uncertainty of the system, which is combined with the dynamic surface control (DSC) technique to avoid the problem of complexity explosion, and its effectiveness is verified in the application of autopilot on marine crafts. In Peng et al. (2014), a distributed adaptive synchronization controller is proposed to provide good decoupling between the observer and controller design of a nonlinear multi-agent system, and to ensure that the state of each agent has bounded residuals. In Li et al. (2022), an RBFNN approximator was adopted to estimate the uncertainties, and by introducing the minimum learning parameter technique to minimize the computational burden associated with neural network weight updates. The NNs were combined with the described performance control when the dynamics were unmodeled (Shen et al., 2020), to guarantee all the followers asymptotically synchronized to the leader, and the synchronization errors within a prescribed level. In Chen et al. (2020), NNs were employed to approximate the unknown external disturbances and uncertain hydrodynamics of unmanned surface vessels, and an adaptive trajectory tracking controller with guaranteed transient performance was developed.

Besides the above model-related factors affecting the performance of the controller, safety is another important factor to be considered in practical application problems (Yang et al., 2022). In containment control problems, the topology of the system determines the convergence positions of followers (Wang et al., 2014). In practical problems, the follower is an entity with a certain volume rather than an idealized prime model, and the resulting collision problem within the system also constrains the translation of theory into practice. The collision avoidance and obstacle avoidance problem of a single vessel can be solved by the artificial potential field method (Liu et al., 2020; Mu and Peng, 2022) or other collision avoidance algorithm (Lu et al., 2022), but for the large-scale cooperative system, due to the network communication load and computational capacity constraints, this method is not very effective, and may even cause the system to be stuck due to repeatedly triggering the collision avoidance conditions. Therefore, considering system safety, it is necessary to seek a reasonable way to reconfigure the system topology from the perspective of the followers' convergence positions. That is to say, through some reasonable topology reconfiguration method, these topologies that may adversely affect the system are reconfigured to achieve specific control objectives such as collision avoidance, accelerating or decelerating the system convergence rate, and maintaining the system communication continuity (Mikulski et al., 2012; Haus et al., 2014; Griparic et al., 2022).

Inspired by the aforementioned studies, in this paper, based on the controller designed in Peng et al. (2014) which takes into account disturbances and model uncertainties with NNs, a synchronized control framework for a containment control system with topology reconfiguration mechanism is proposed, and the process of containment control is converted into the tracking of a reference signal for each vessel on its respective target heading by means of the LOS guidance method. With the proposed hierarchical control framework, the communication topology of the follower layer can be reconfigured to enable the transformation of follower formation and dispersion Securing the system throughout its operation.

## 2. Materials and methods

### 2.1. Graph theory

Consider a system G=(V,E) composed by *n* agents, where $V=\left(v_{1},v_{2},...,v_{n}\right)$ represents the set of vertices, $E=\left\{\left(v_{i},v_{j}\right)|v_{i},v_{j}\in V\right\}$ denotes the set of edges, representing the link between every two vertices. The link between every two vertices in the graph is defined by the adjacency matrix $A\in {\mathbb{R}}^{n\times n}$, where *a*_*ij*_ = 1 means that there is an edge directed to *v*_*i*_ from *v*_*j*_, otherwise *a*_*ij*_ = 0. The degree matrix $D=diag\left(d_{1},d_{2},...d_{n}\right)$ is a diagonal matrix where $d_{i}=\overset{n}{\underset{j=1}{\sum }}a_{ij}$ denotes the degree of vertex *i*. Another adjacency matrix $A_{0}\in {\mathbb{R}}^{n\times n}$ is defined to describe the relationship between the virtual leader and the other agents, denoted $A_{0}=diag\left(a_{i0}\right)$, where *a*_*i*0_ = 1 indicates that there is an edge directed from the virtual leader to *v*_*i*_, otherwise *a*_*i*0_ = 0. In our study, a matrix denotes the whole system, which is given as $H=L+A_{0}$.

The Laplacian matrix is defined

(1) $$\begin{array}{l} L=D-A \end{array}$$

where

(2) $$\begin{array}{l} l_{ij}=\{\begin{array}{c} \overset{n}{\underset{j=1}{\sum }}a_{ij},i=j\\ -a_{ij},i\neq j \end{array} \end{array}$$

In ascendental order, the eigenvalues of the Laplace matrix are as follows

$$\lambda_{1}\leq \lambda_{2}\leq \ldots \leq \lambda_{n}$$

The second minimal eigenvalue of the Laplacian matrix, λ_2_, is also known as the algebraic connectivity. For undirected graph, when and only when λ_2_ > 0, the graph is connected (Fiedler, 1973).

### 2.2. Containment control

For a containment control system consisting *m* leaders as well as *n* − *m* followers, the Laplacian matrix can be expressed as

(3) $$\begin{array}{l} L=\left[\begin{array}{cc} 0_{m\times m} & 0_{m\times \left(n-m\right)}\\ L_{1} & L_{2} \end{array}\right] \end{array}$$

where $L_{1}\in {\mathbb{R}}^{\left(n-m\right)\times m}$, $L_{2}\in {\mathbb{R}}^{\left(n-m\right)\times \left(n-m\right)}$.

To analyze the containment control of a system, Lemma 1 is given:

**Lemma 1**. Assume that the communication digraph *G* has a directed spanning forest. The sum of each row of $-L_{2}^{-1}L_{1}$ is 1 and the element of $-L_{2}^{-1}L_{1}$ is positive if and only if the *i*th leader has a directed path to the *j*th follower (Shan et al., 2021).

Then, the position vectors of leaders as well as followers are denoted as

(4) $$\begin{array}{l} \left[\phi_{m+1},\phi_{m+2},...,\phi_{n}\right]=\left(-L_{2}^{-1}L_{1}⊗I_{2}\right)\left[\phi_{1},\phi_{2},...,\phi_{m}\right] \end{array}$$

where $\phi_{i}={\left[X_{i},Y_{i}\right]}^{T}$ denotes position vectors within *X*_*E*_ − *Y*_*E*_ coordinate system.

### 2.3. System modeling

The kinematic model of the *i*th unmanned surface vessel is given as

(5) $$\begin{array}{l} \left[\begin{array}{c} {\dot{X}}_{i}\\ {\dot{Y}}_{i}\\ {\dot{\psi }}_{i} \end{array}\right]=\left[\begin{array}{ccc} \cos\left(\psi_{i}\right) & -\sin\left(\psi_{i}\right) & 0\\ \sin\left(\psi_{i}\right) & \cos\left(\psi_{i}\right) & 0\\ 0 & 0 & 1 \end{array}\right]\left[\begin{array}{c} u_{i}\\ v_{i}\\ r_{i} \end{array}\right] \end{array}$$

where ${\left[X_{i},Y_{i}\right]}^{T}$ is the position vector within *X*_*E*_ − *Y*_*E*_ coordinate system, ψ_*i*_ denotes the heading, ${\left[u_{i},v_{i},r_{i}\right]}^{T}\in {\mathbb{R}}^{3}$ denotes the linear velocity vector and the angular velocity vector of the *i*th unmanned surface vessel.

To make the controller design simpler, Assumption 1 is given.

**Assumption 1**. For each vessel in control system, the linear velocity *u* is a positive constant and the angular velocity *v* = 0.

For marine crafts, which usually have separate speed controllers for speed control, it is a reasonable assumption that *u* is a constant. During manipulation, the motion in the direction of the linear velocity is small relative to the motion in the other directions so it can be ignored (Li et al., 2009).

Considering a containment control system consisting of several unmanned surface vessels, the dynamics of each is stated as (Li et al., 2010)

(6) $$\begin{array}{l} \left[\begin{array}{c} {\dot{\psi }}_{i}\\ {\ddot{\psi }}_{i} \end{array}\right]=\left[\begin{array}{cc} 0 & 1\\ 0 & 0 \end{array}\right]\left[\begin{array}{c} \psi_{i}\\ {\dot{\psi }}_{i} \end{array}\right]+\left[\begin{array}{c} 0\\ K/T \end{array}\right]\left[\omega_{i}-F\left({\dot{\psi }}_{i}\right)\right] \end{array}$$

where ψ_*i*_, ${\dot{\psi }}_{i}$, and ω_*i*_ representatively denote the heading, the rate of heading, and the heading moment of *i*th vessel, K and T are parameters that represent the maneuverability of marine crafts. $F\left({\dot{\psi }}_{i}\right)$ is an unknown nonlinear function of ${\dot{\psi }}_{i}$, which is estimated by following equation

(7) $$\begin{array}{l} F\left({\dot{\psi }}_{i}\right)=b_{i1}{\dot{\psi }}_{i}+b_{i2}{\dot{\psi }}_{i}^{3}+b_{i3}{\dot{\psi }}_{i}^{5}+\cdots \end{array}$$

### 2.4. Control methods

**Figure 1:**
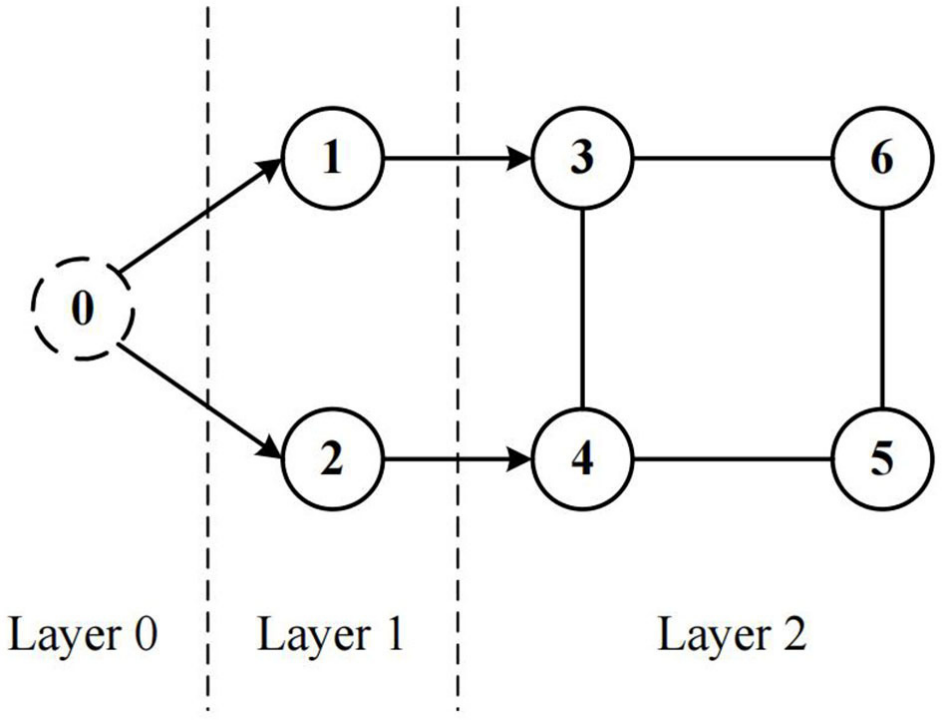
System communication topology.

In the context of the cooperative containment control problem guided by a virtual leader, the system in terms of topology could be segmented into three layers, as shown in Figure 1. Layer 0 is the virtual leader layer, which gives the reference tracking signal. Layer 1 is the real leader layer, where all the real leaders track the reference signal through the virtual leader. Layer 2 is the follower layer, according to the real-time position of the real leaders, the respective reference trajectory point can be calculated for each follower, and the path-following process is converted as a heading tracking process through the LOS guidance, so as to achieve the cooperative containment control.

A topology reconfiguration strategy based on communication measurements is also incorporated in the second layer, to reconfigure the communication topology in time so as to maintain the performance level of the system.

**Figure 2:**
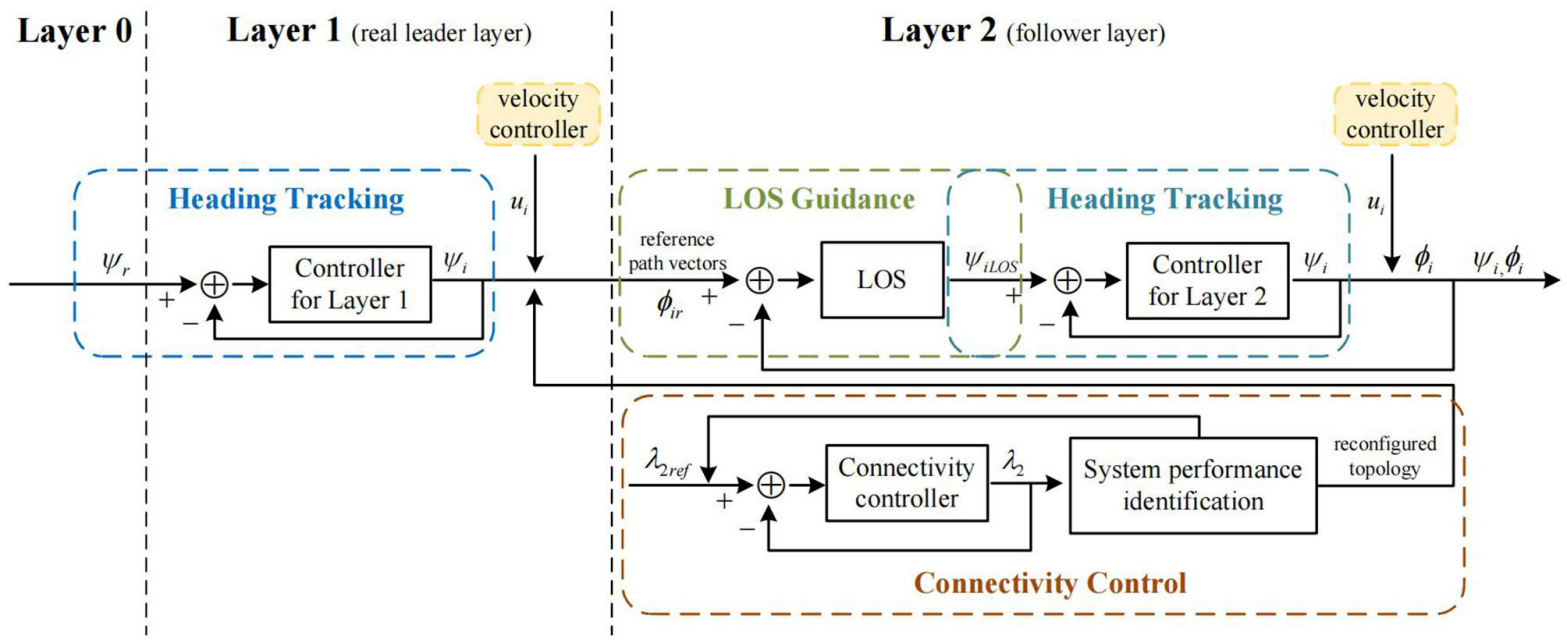
The hierarchical control framework in this paper.

**Figure 3:**
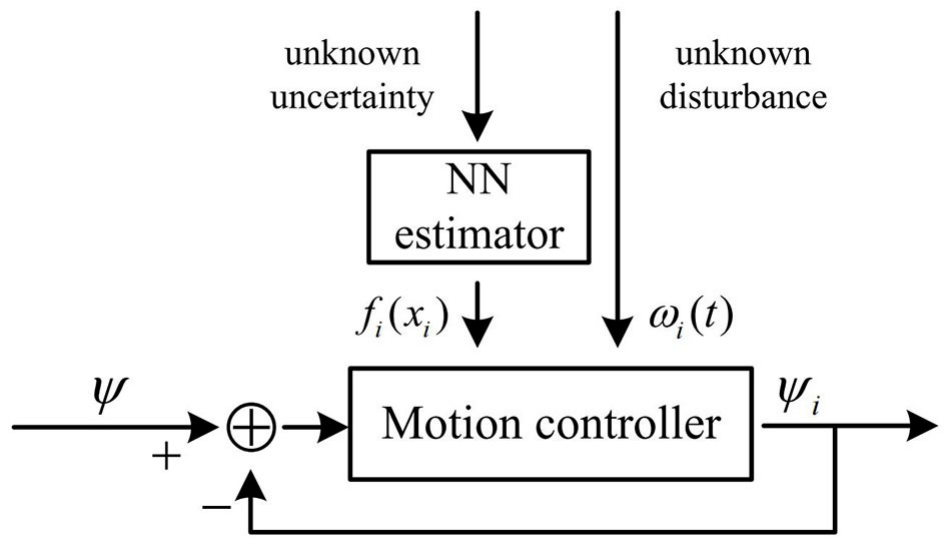
The controller for Layer 1 and Layer 2.

The control method of the system proposed in this paper is described in three steps: (i) the real leader's tracking of the reference heading, (ii) the follower's tracking of the respective reference trajectory under containment control, (iii) the follower's tracking of the new reference trajectory after a change of the system's communication topology. The hierarchical control framework proposed in this paper is shown in Figure 2. The controller for Layer1 and Layer 2 are the same in form, as shown in Figure 3, wherein the controller for Layer 1, ψ denotes ψ_*r*_, and in the controller for Layer 2, ψ denotes ψ_*iLOS*_.

#### 2.4.1. Design of LOS guidance law based on containment control system

By the real leaders' tracking of the reference heading, combined with the kinematic equations given as Eq. (5), and with Eq. (4), the followers' desired positions could be calculated timely. For the goal of achieving containment control, the path-following process is converted as a time-varying heading tracking process by means of LOS guidance.

To design the tracking controller, the Serret-Frenet coordinate system is usually used, which is established by taking the current position of the marine craft (*X, Y*) projected on the reference path (*X*_*d*_(ω), *Y*_*d*_(ω)) as the origin, where ω is the path parameter variable. The reference heading angle is

(8) $$\begin{array}{l} \psi_{p}=\arctan\frac{{\dot{Y}}_{p}\left(\omega \right)}{{\dot{X}}_{p}\left(\omega \right)} \end{array}$$

where Ẏ_*p*_(ω) = *dY*_*p*_(ω)/*dω*, Ẋ_*p*_(ω) = *dX*_*p*_(ω)/*dω*, the changing rate of the path parameter variable satisfying

(9) $$\begin{array}{l} \dot{\omega }=U/\sqrt{{\dot{X}}_{p}^{2}\left(\omega \right)+{\dot{Y}}_{p}^{2}\left(\omega \right)}>0 \end{array}$$

where $U=\sqrt{u^{2}+v^{2}}$ denotes the speed of navigation.

**Figure 4:**
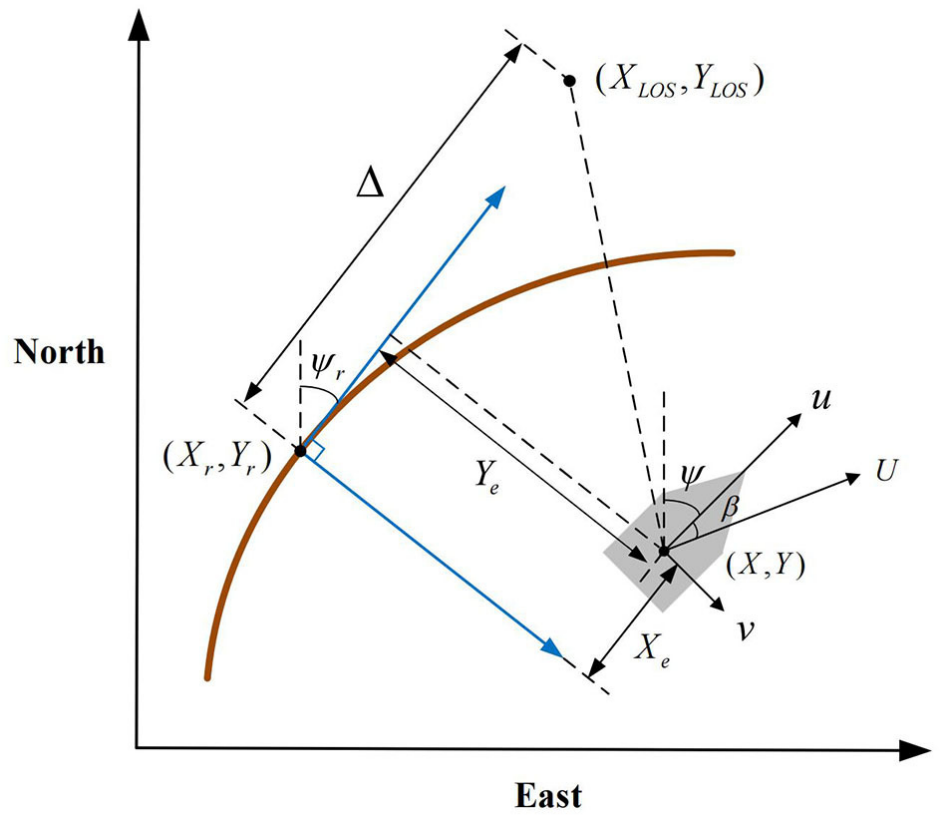
The schematic of LOS guidance.

The schematic of LOS guidance is given in Figure 4, the LOS guidance law based on forward-looking distance is expressed as

(10) $$\begin{array}{l} \psi_{LOS}=\psi_{p}+\arctan\left(-Y_{e}/\Delta \right) \end{array}$$

where Δ is the forward-looking distance. Thus the reference heading angle of followers is

(11) $$\begin{array}{l} \psi_{d}=\psi_{LOS}-\beta \end{array}$$

where $\beta =\arctan\frac{v}{u}$ reprensents the angle of drift.

From geometrical relations in Figure 1, the error of path tracking is obtained as follows

(12) $$\begin{array}{l} \left[\begin{array}{c} X_{e}\\ Y_{e} \end{array}\right]=\left[\begin{array}{cc} \cos\psi_{p} & \sin\psi_{p}\\ -\sin\psi_{p} & \cos\psi_{p} \end{array}\right]\left[\begin{array}{c} X-X_{r}\\ Y-Y_{r} \end{array}\right] \end{array}$$

where *Y*_*e*_ is the position tracking error, and the desired position $\phi_{ir}={\left[X_{r},Y_{r}\right]}^{T}$ can be calculated by Eq. (4).

For path-following problems where the path parameters are known, the LOS guidance law is stated as the above equation. In this paper, the reference heading is known while the global path is unknown, also, with Assumption 1, the effect of drift angle is not considered. Thus the guidance law for the *i*th follower marine craft in the system can be designed as

(13) $$\begin{array}{l} \begin{array}{c} \psi_{iLOS}=\psi_{p}+\arctan\left(-Y_{ie}/\Delta \right)=\psi_{r}+\arctan\left(-Y_{ie}/\Delta \right)\\ \psi_{id}=\psi_{iLOS}-\beta =\psi_{iLOS},\;i=1,...,n-m \end{array} \end{array}$$

#### 2.4.2. Controller design

Having $x_{i}=\left[\psi_{i},{\dot{\psi }}_{i}\right]$, $f_{i}\left(x_{i}\right)=F\left({\dot{\psi }}_{i}\right)$ and selecting ω_*i*_ = *u*_*i*_+*K*_*r*_*x*_*i*_, in which *K*_*r*_ is a feedback matrix, then the model can be written as

(14) $$\begin{array}{l} \{\begin{array}{c} {ẋ}_{i}=Ax_{i}+B\psi_{r}\\ y_{i}=Cx_{i},\;i\in {\text{L}}_{v}\\ {ẋ}_{i}=Ax_{i}+B\left[u_{i}+f_{i}\left(x_{i}\right)+\omega_{i}\left(t\right)\right]\\ y_{i}=Cx_{i},\;i\in {\text{L}}_{r},\text{F} \end{array} \end{array}$$

where

(15) $$\begin{array}{l} A=\left[\begin{array}{cc} 0 & 1\\ 0 & 0 \end{array}\right]+BK_{r} \end{array}$$

is Hurwitz, and

(16) $$\begin{array}{l} B=\left[\begin{array}{c} 0\\ K/T \end{array}\right] \end{array}$$

**L**_*v*_, **L**_*r*_ and **F** denote the virtual leaders, the real leaders, and followers respectively, let **L**_*v*_ = {0}, **L**_*r*_ = {1, 2, ..., *m*}, **F** = {*m* + 1, *m* + 2, ..., *n*}. ψ_*r*_(*t*) is the reference input, *u*_*i*_ is the control input, *f*_*i*_(*x*_*i*_) is the unknown uncertainty and ω_*i*_(*t*) is disturbance which is unknown but bounded.

According to Stone (1948), *f*_*i*_(*x*_*i*_) is approximated with the neural networks, which is as follows

(17) $$\begin{array}{l} f_{i}\left(x_{i}\right)=W_{i}^{T}\varphi_{i}\left(x_{i}\right)+\varepsilon_{i},\;\forall x_{i}\in \Pi \end{array}$$

where $W_{i}\in {\mathbb{R}}^{s}$ denotes the ∈ℝ^*s*×*m*^ satisfying ||*W*_*i*_|| ≤ *W*_*M*_ is a constant real matrix which denotes the ideal neural network (NN) weight matrix, φ_*i*_(·) is a known basis function, ε_*i*_ is the neural network approximation error satisfying ||ε_*i*_|| ≤ ε_*M*_.

(1) Controller for vessels in Layer 1

As given in Zhang et al. (2011), the following control protocol is considered:

(18) $$\begin{array}{l} u_{in}=cK\left(\underset{j\in N_{i}}{\sum }a_{ij}\left(x_{i}-x_{j}\right)+a_{i0}\left(x_{i}-x_{0}\right)\right) \end{array}$$

where *c* ∈ ℝ is a coupling gain, *K* = −*B*^*T*^*P*^−1^ is the feedback, of which P is a positive definite solution of linear matrix inequality (LMI)

(19) $$\begin{array}{l} A^{T}P+PA+Q-PBB^{T}P\leq 0 \end{array}$$

where *Q* ∈ ℝ^*n*×*n*^ is positive definite.

Considering the uncertain nonlinear dynamical terms in the model, an adaptive control term *u*_*iad*_ is given as

(20) $$\begin{array}{l} u_{iad}={\hat{W}}_{i}^{T}\varphi_{i}\left(x_{i}\right) \end{array}$$

where ${\hat{W}}_{i}$ is the estimation of *W*_*i*_. Thus the control law can be written as

(21) $$\begin{array}{l} u_{i}=u_{in}-u_{iad} \end{array}$$

Combined with Figure 1 and the aforementioned, the object of the control law (13) implementation is a directed graph. According the results given in Peng et al. (2014), the update law of ${\hat{W}}_{i}$ should be chosen as

(22) $$\begin{array}{l} {\dot{\hat{W}}}_{i}=\Gamma_{W_{i}}\left[\tau_{i}\left(d_{i}+a_{i0}\right)\varphi_{i}\left(x_{i}\right)e_{i}^{T}PB-k_{W}{\hat{W}}_{i}\right] \end{array}$$

where Γ_*W*_*i*__, *k*_*W*_ are constant to be designed, *e*_*i*_ and τ_*i*_ is respectively given as follows

(23) $$\begin{array}{l} \begin{array}{c} e_{i}=\sum_{j\in N_{i}}a_{ij}\left(x_{i}-x_{j}\right)+a_{i0}\left(x_{i}-x_{0}\right)\\ T=diag\left(\tau_{i}\right)=diag\left(1/q_{i}\right)\\ q={\left[q_{1},...,q_{i}\right]}^{T}=H^{-1}1,\;i=1,...m \end{array} \end{array}$$

then, all signals in the closed-loop network can be uniformly ultimately bounded.

(2) Controller for vessels in Layer 2

**Figure 5:**
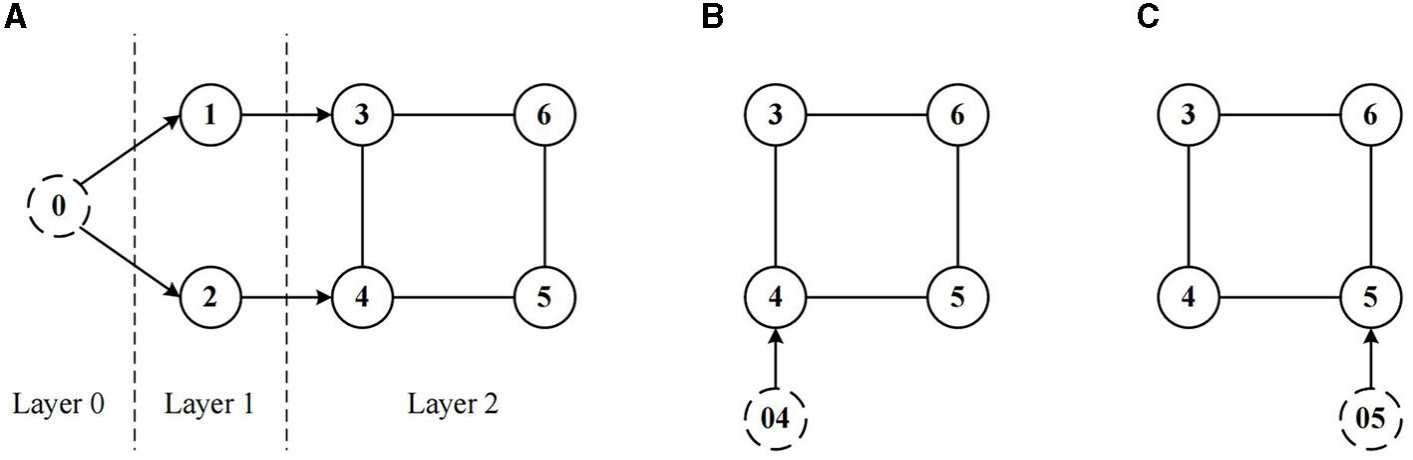
Communication topology of followers in Layer 2. **(A)** System topology, **(B)** communication topology of follower 4, and **(C)** communication topology of follower 5.

From the former analysis, in the follower layer (i.e. Layer 2) the process of tracking the reference trajectory by each follower could be considered as the heading tracking process led by the virtual leader. Thus, in Layer 2, each follower can be also represented as follows, as shown in Figure 5, where *x*_0*i*_ denotes the virtual leader for the *i*th follower. With this form, the follower receives control signals not directly from the leader, but from its corresponding virtual leader and its neighbors.

Thus, in Layer 2, the controller is designed as

(24) $$\begin{array}{l} \begin{array}{c} u_{i}=u_{in}-u_{iad}\\ u_{in}=cK\left(\sum_{j\in N_{i}}a_{ij}\left(x_{i}-x_{j}\right)+\left(x_{0i}-x_{0}\right)\right)\\ u_{iad}={\hat{W}}_{i}^{T}\varphi_{i}\left(x_{i}\right) \end{array} \end{array}$$

where the update law of ${\hat{W}}_{i}$ is

(25) $$\begin{array}{l} {\dot{\hat{W}}}_{Fi}=\Gamma_{W_{Fi}}\left[\tau_{i}\left(d_{i}+1\right)\varphi_{i}\left(x_{i}\right)e_{i}^{T}PB-k_{FW}{\hat{W}}_{i}\right],\;i=1,...n-m \end{array}$$

where Γ_*W*_*Fi*__, *k*_*FW*_ are constant to be designed. According to the proof in Peng et al. (2014), it can be also obtained that all signals in the closed-loop network can be uniformly ultimately bounded.

(3) Connectivity control

The autopilot of the vessel, as an assistant tool in the process of navigation, if timely changes can be made based on the current or possible conditions when cooperating, the safety performance of the system and the efficiency of cooperation will be greatly improved. Several classical cases of topology reconfiguration are to change the converging positions of followers so as to avoid collision within the system, or to maintain the system connectivity when the communication equality is poor.

A topology reconfiguration algorithm was given by Griparic et al. (2022), which enabled the algebraic connectivity of the system to reach the desired value through its proposed method of adding/removing links. The inputs of the topology reconfiguration algorithm are the initial local adjacency matrix of each marine craft $A_{F}^{l}\left(0\right)$(F means that the marine craft is a follower and *l* = 1, 2, 3, ..., *n* − *m* represents the index of marine craft), desired level of system performance λ_2*ref*_, and user-defined value *K*_λ_2__. The connectivity controller can be stated as follows

(26) $$\begin{array}{l} e_{\lambda_{2}}^{l}\left(k\right)=\lambda_{2ref}-\lambda_{2}^{l}\left(k\right) \end{array}$$

the parameter *K*_λ_2__ > 0 is to be determined so as to have

(27) $$\begin{array}{l} |e_{\lambda_{2}}^{l}\left(k\right)|<K_{\lambda_{2}},\;k\to \infty \end{array}$$

Combined with the RNN-based system performance identification model proposed by Liu et al. (2023), the features of the containment control system can be extracted as the following matrix, which is considered as the input of the RNN-based performance identification model

(28) $$\begin{array}{l} \Theta_{Input}=\left[\begin{array}{ccccccc} \theta_{1} & \theta_{2} & \cdots & \cdots & \theta_{i} & \cdots & \theta_{n-m}\\ d_{1} & d_{2} & \cdots & \cdots & d_{i} & \cdots & d_{n-m}\\ d_{N_{1}} & d_{N_{2}} & \cdots & \cdots & d_{N_{i}} & \cdots & d_{N_{n-m}}\\ d_{N_{1}}^{N} & d_{N_{2}}^{N} & \cdots & \cdots & d_{N_{i}}^{N} & \cdots & d_{N_{n-m}}^{N} \end{array}\right] \end{array}$$

where Θ_*Input*_ represents the input to the NN where the first row composed of ones and zeros describes the connection relationship between the leader and follower agents in the system, θ_*i*_ = 1, *i* ∈ [1, *n* − *m*] indicates that the leader is connected to the *i*th follower while 0 denotes no connection. The remaining three rows are vectors transformed from the node degree relativity matrix, *d*_*i*_ denotes the degree of the *i*th follower, $d_{N_{i}}$ denotes the sum of degrees of the neighbor set of the *i*th follower, and $d_{N_{i}}^{N}$ denotes the sum of degrees of the neighbor set nodes of the *i*th follower's neighbors.

The output is the performance indicators of the containment control system, given as

(29) $$\begin{array}{l} \begin{array}{c} \Theta_{Output}=\left[\begin{array}{c} \lambda_{2}\\ \sigma \left(X_{FP}\right) \end{array}\right] \end{array} \end{array}$$

where λ_2_ is the algebraic connectivity of the network in Layer 2, and $\sigma \left(X_{FP}\right)$ is used to describe the dispersion of converging positions of followers in the convex hull spanned by all leader.

Through this RNN-based performance identification model, the system topology with good collision-avoiding performance can be selected. Thus the reconfiguration of topology is finished.

To integrate topology reconfiguration with system control, it is assumed that the communication between each marine craft is continuous. Thus, topology reconfiguration can be done within a very short period of time which is represented as a time-varying Laplacian matrix in the control system, expressed as

(30) $$\begin{array}{l} L_{c2}\left(t\right)=\{\begin{array}{cc} L_{c0}, & 0<t<t_{R}\\ L_{cR}, & t_{R}\leq t \end{array} \end{array}$$

where *L*_*c*0_ denotes the initial topology of Layer 2, and *L*_*cR*_ denotes the reconfigured topology of Layer 2.

**Figure 6:**
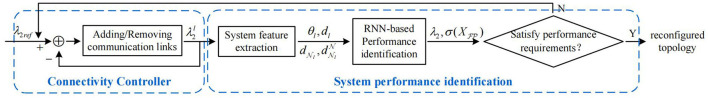
The framework of connectivity control in Layer 2.

Based on the premise of the containment control problem, only the topology of Layer 2 is reconfigured in this paper. The framework of connectivity control is shown in Figure 6, where the $\lambda_{2}^{l}$ denotes the algebraic connectivity after each processing, and the identification object of system performance is whether the dispersion of followers is large enough so as to keep a safe distance for each two followers. The system performance identification module is a determination of the reconfigured topology output by the connectivity controller. If the determination is ‘Yes”, the reconfigured topology satisfying the system performance requirements is obtained and output. If not, the control signal will be transmitted back to the first step which is to input λ_2*ref*_ and start another round of topology reconfiguration.

## 3. Simulation results and discussions

**Figure 7:**
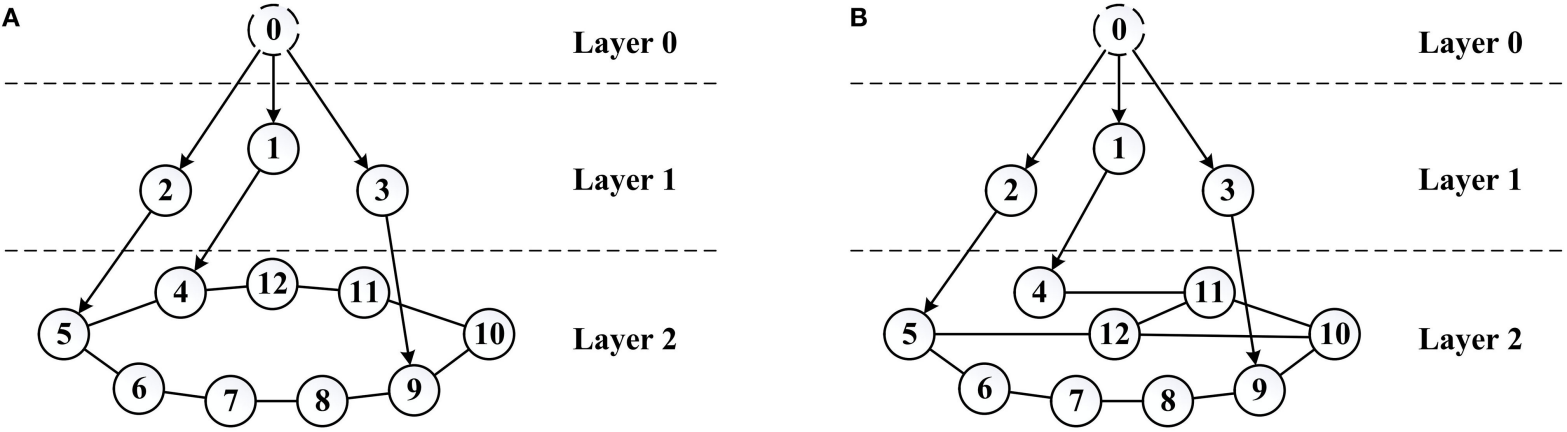
System communication topology. **(A)** Initial system topology and **(B)** reconfigured system topology.

The simulation is implemented to validate the effect of the control law (21) and (24), LOS guidance law (13), as well as the connectivity control algorithm designed in Section 3.2.3. Considering a multiple unmanned surface vessel system (6) with 1 virtual leader, 3 real leaders, and 9 followers, whose initial communication topology is shown in Figure 7A.

The initial position vectors of real leaders are given as $\phi_{1}={\left[0,100\right]}^{T}$, $\phi_{2}={\left[0,-100\right]}^{T}$ and $\phi_{3}={\left[100,0\right]}^{T}$. The initial position vectors of followers are given as $\phi_{4}={\left[0,300\right]}^{T}$, $\phi_{5}={\left[0,250\right]}^{T}$, $\phi_{6}={\left[0,200\right]}^{T}$, $\phi_{7}={\left[0,150\right]}^{T}$, $\phi_{8}={\left[0,100\right]}^{T}$, $\phi_{9}={\left[0,-150\right]}^{T}$, $\phi_{10}={\left[0,-200\right]}^{T}$, $\phi_{11}={\left[0,-250\right]}^{T}$ and $\phi_{12}={\left[0,-300\right]}^{T}$. The speed of each marine crat is *u* = 8*m*/*s*, *v* = 0. Forward-looking distance is Δ = 150*m*.

The parameters are given as *b*_*i*1_ = 0.1256, *b*_*i*2_ = 0.3576, *b*_*i*3_ = 0.0278. The coupling gain *c* = 30, the feedback matrix $K_{r}={\left[-1-40\right]}^{T}$, and the ordered reference input ψ_*r*_ is given as

(31) $$\begin{array}{l} \psi_{r}\left(t\right)=\{\begin{array}{cc} 0, & 0<t<500\\ 0.25, & 500\leq t<1000\\ 0, & 1000\leq t<1500\\ 0.25, & 1500\leq t<2000\\ 0, & 2000\leq t \end{array} \end{array}$$

When using the neural network for estimation, to make the estimation better, the adaptive parameters of the NN should take larger values. Thus taking Γ_*W*_*i*__ = 100 and Γ_*W*_*Fi*__ = 100. *k*_*W*_ and *k*_*FW*_ are taking as *k*_*W*_ = *k*_*FW*_ = 0.8. φ_*i*_(*x*_*i*_) is given as $\varphi_{i}\left(x_{i}\right)=1/e^{-x_{i}}$.

Letting $Q=\left[\begin{array}{cc} 0.1 & 0\\ 0 & 0.1 \end{array}\right]$, through solving (19), one can obtain that

(32) $$\begin{array}{l} K=\left[-0.0661,-0.6920\right],\;P=\left[\begin{array}{cc} 3.3632 & 26.4333\\ 26.4333 & 276.7941 \end{array}\right] \end{array}$$

**Figure 8:**
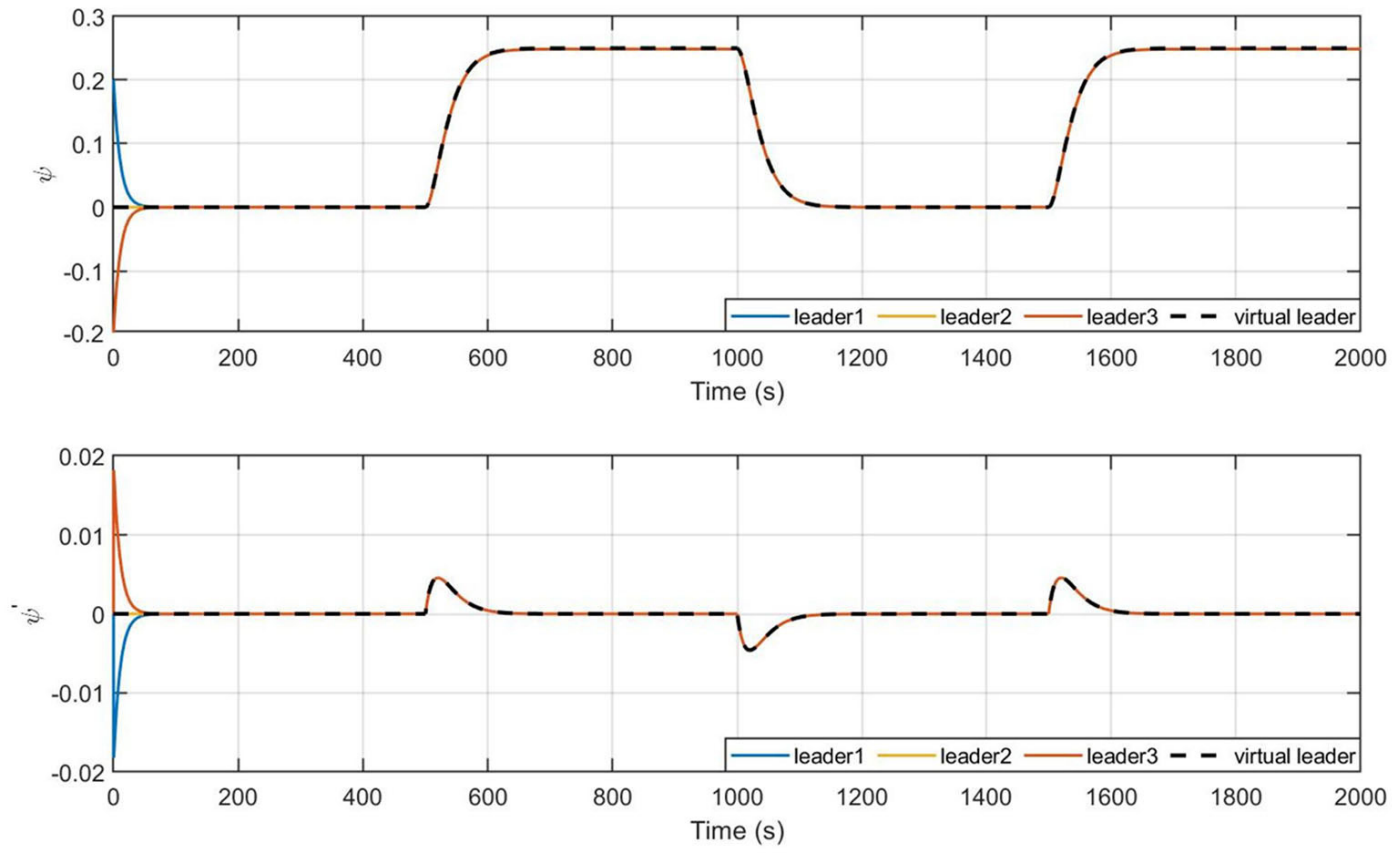
State profile of leaders.

**Figure 9:**
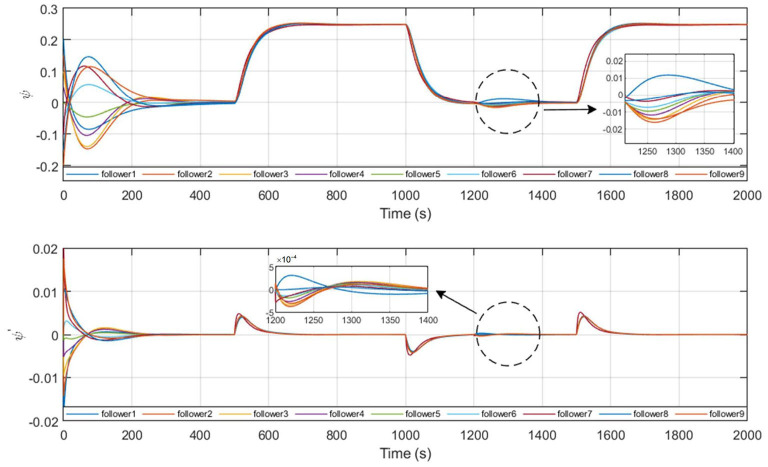
State profile of followers.

Figures 8, 9 give the state profile of real leaders and followers, in which the real leaders track well on the reference signal given by the virtual leader, and with the LOS guidance law, the followers also achieve tracking of the targeted heading, thus converging into the convex hull spanned by leaders.

**Figure 10:**
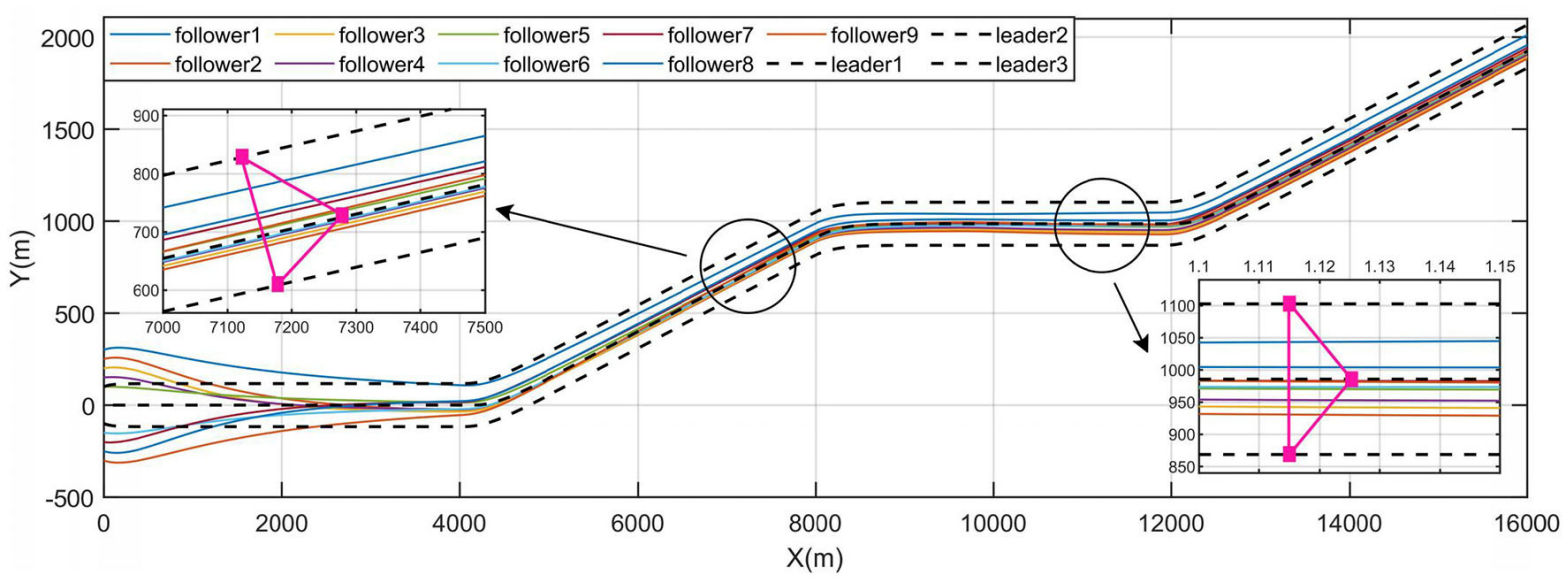
Path profile of leaders and followers.

The path profile is given in Figure 10, in which can be observed that with the initial system communication topology, the converging positions of the followers are very tight. Most of the marine crafts are not distant enough from each other to meet the safety distance requirement, in which case the risk of collision within the system will be greatly increased. Thus, combined with the connectivity control method and the system performance identification model stated in Section 3.2.3, a reference value λ_2*ref*_ = 0.4 is chosen as the desired performance index of Layer 2 and *K*_λ_2__ is taken as *K*_λ_2__ = 0.1, which is used to reconfigure the communication topology of Layer 2 so as to disperse the converging positions of followers. The reconfigured system topology is shown in Figure 7B, accordingly, λ_2_ = 0.4131.

The process of topology reconfiguration is performed as *t* = 1, 200*s*. It is presented in Figure 9 that the response of the control system when the reference path tracked by the followers is changed after the topology reconfiguration is completed.

## 4. Conclusion

This paper investigated the containment control of multiple unmanned surface vessels with NN control via reconfigurable hierarchical communication topology. A hierarchical control framework was proposed, so as to transform the containment control problem of followers to the synchronization of reference heading tracking, which is realized with the LOS guidance law. In the control system, the NNs are adopted to consider the uncertainty. In the follower layer, a connectivity controller with a topology reconfiguration mechanism was embedded to reconfigure the communication topology, which is used to improve the safety of the system when operating. It was shown in the simulation results that with this hierarchical control framework, the real leader and the virtual leader, and the follower and the real leader all achieved well-tracking, and the controller also achieved the tracking of the new reference signal without significant oscillations of control output when the communication topology of the follower layer was changed. In this case, the converging positions of the followers were dispersed throughout the system when tracking the reference heading during containment control of multiple marine crafts, which greatly reduced the risk of collisions within the system.

## Data availability statement

The raw data supporting the conclusions of this article will be made available by the authors, without undue reservation.

## Author contributions

WL: Conceptualization, Formal analysis, Methodology, Writing—original draft. FT: Conceptualization, Methodology, Validation, Writing—review and editing. HX: Software, Writing—original draft. CW: Writing—original draft.
